# Editoral

**Published:** 1881-02

**Authors:** 


### HOMŒOPATHIC PHYSICIAN.

A MONTHLY JOURNAL OF MEDICAL SCIENCE.

"If our school ever gives up the strict inductive method of Hahnemann, we are lost, and deserve to be mentioned only as a caricature, in the history of medicine." — CONSTANTINE HERING.

Vol. I. FEBRUARY, 1881. No. 2.

### EDITORIAL.

It has pleased a kind *friend* to take exception to our humble salutatory. To him there seems to be no danger threatening the continuance or purity of homœopathy. As there may be many, who likewise think we cry "wolf" when there is no wolf, we will briefly review a few of the insidious changes now going on, which jeopardize the future of homœopathy.

First, allow us to say that the wolf hidden in the sheep's skin is the most dangerous of all foes. So, the most hurtful enemies homœopathy has to-day, are some of its *adherents*—so-called. These persons injure homœopathy in many ways. Their eclectic and unsuccessful methods of practising — being *called* homœepathic—detract from its reputation as a curative system˙ Their peculiar morals, professing one thing and practising another, give homœopathy a bad name, compelling honest men to declare that "for a man to practise homœopathy *at present* necessitates that he be either ignorant, foolish or knavish; that is, if it be knavish to live a lie." In consequence of these things, the proper and natural growth of homœopathy is retarded. The honest inquirer turns away in disgust; the invalid, racked and torn by the harsh methods of allopathy, sees no relief in mongrelism.

The causes of this retrograde movement in our school are many; a few of the most prominent, we will mention. Firstly, our medical schools do not teach *pure* homœopathy. Instead of teaching the system as Hahnemann taught it, they wander into pathological and other equally erroneous deviations; instead of teaching the plain life-saving truth as demanded by the law of the similars, they dress it in fancy garbs, that they may masquerade as "scientific" physicians. In consequence of these "fatal errors" the works of Hahnemann are unknown to most graduates. As a bookseller informed the writer, they are "practically out of print," because there is no demand for them! Less than one thousand copies of the "Organon" have been sold to a profession said to number six thousand! Who nowadays buys Hahnemann's "Chronic Diseases"? It is idle to reply that Hahnemann's writings are sufficiently well taught by lecture, for it can not be done. Our learned professors might, at least, teach Hahnemann's "Organon" until they have leisure to write something better.

Another grievous source of injury to homœopathy, and a natural sequence of bad teaching, are many of the text books now published; works which serve only to corrupt our friends and furnish ammunition to our foes. There are many books professing to teach and illustrate homœopathy which are totally unfit for the purpose; these penny-a-liners may do little injury to the older and more settled members of the profession, for they know better; but to those of less settled opinion, they do incalculable injury. If the beginner takes one of these works as his guide in practice, he is almost sure to fail in his first venture; then he stumbles from bad to worse. From the single remedy, perhaps in low potency, he alternates his drugs, and, getting no good results from such doings, he promptly proclaims homœopathy insufficient, and resorts to quinia, palliatives, tonics, cathartics, etc., all of which is credited by suffering patients and watchful adversaries to homœopathy, and we are called deceitful knaves, etc. Says an allopath, "Allopathy

Homœopathy, Hydropathy—can any one or all of these be true ¿ Some doctors hold to one, some to the other. Occasionally you hear of an individual practicing all three! To do this last *honestly* is a very clever thing!" (Dickson).

A third source of contamination, for our school, are articles which continually appear in our journals and disseminate their fatal germs throughout the profession. Articles which desplay gross ignorance of homœopathy, allopathy, and every other kind of medicine. "Our literature became such an admixture of truth and falsity, that only the most astute could sift them," (H. M., p. 693, Nov. 1880). When colleges, authors and journals, are continually belching forth their deadly poison can we wonder that the rank and file desert the law and depart from "the strict inductive method of Hahnemann"? When a professor in a homœopathic (?) college gives morphia to insure "needful rest," or a president of a state homœopathic medical society gives bichr. of potash, in massive doses, to change the name of a disease, is there no danger of a departure from "the strict inductive method of Hahnemann"? When an ex-president of The American Institute of Homœopathy proclaims that "one who only occasionally prescribes homœopathically is a homœopathist," or when a president of that noble body openly and proudly boasts of his exhibition of cathartics, etc., is there no departure from "the strict inductive method of Hahnemann"? As the editor of *The Hahnemannian Monthly* truly says, "we have wandered far from the principles of true homœopathy, and unless we return, our noble art will be buried in electicism."* "Truth is law" and our law is truth!

These wanderings "from the principles of true homœopathy," to which we have briefly alluded, can not be honestly denied; still less can it be claimed that they do not jeopardize the future of homœopathy. Shall we "be buried in electicism" to "live in the history of medicine, as a caricature?"

* Every one should read this admirable editorial, from which we have quoted. It is to be found in *The H. Monthly* for Nov '80 p. 693

